# Differential effect of obesity on bone mineral density in White, Hispanic and African American women: a cross sectional study

**DOI:** 10.1186/1743-7075-2-9

**Published:** 2005-04-07

**Authors:** Jonathan P Castro, Linda A Joseph, John J Shin, Surender K Arora, John Nicasio, Joshua Shatzkes, Irina Raklyar, Irina Erlikh, Vincent Pantone, Gul Bahtiyar, Leon Chandler, Lina Pabon, Sara Choudhry, Nilofar Ghadiri, Pramodini Gosukonda, Rangnath Muniyappa, Hans von-Gicyzki, Samy I McFarlane

**Affiliations:** 1 Department of Medicine, State University of New York Downstate Medical Center, Brooklyn, New York 11203, USA; 2 Division of Endocrinology, Diabetes and Hypertension, State University of New York Downstate Medical Center, Brooklyn, New York 11203, USA; 3 Scientific Computing and Statistics Center, State University of New York Downstate Medical Center, Brooklyn, New York 11203, USA

## Abstract

Osteoporosis is a major public health problem with low bone mass affecting nearly half the women aged 50 years or older. Evidence from various studies has shown that higher body mass index (BMI) is a protective factor for bone mineral density (BMD). Most of the evidence, however, is from studies with Caucasian women and it is unclear to what extent ethnicity plays a role in modifying the effect of BMI on BMD.

A cross sectional study was performed in which records of postmenopausal women who presented for screening for osteoporosis at 2 urban medical centres were reviewed. Using logistic regression, we examined the interaction of race and BMI after adjusting for age, family history of osteoporosis, maternal fracture, smoking, and sedentary lifestyle on BMD. Low BMD was defined as T-score at the lumbar spine < -1.

Among 3,206 patients identified, the mean age of the study population was 58.3 ± 0.24 (Years ± SEM) and the BMI was 30.6 kg/m^2^. 2,417 (75.4%) were African Americans (AA), 441(13.6%) were Whites and 348 (10.9%) were Hispanics. The AA women had lower odds of having low BMD compared to Whites [Odds ratio (OR) = 0.079 (0.03–0.24) (95% CI), p < 0.01]. The odds ratio of low BMD was not statistically significant between White and Hispanic women. We examined the interaction between race and BMD. For White women; as the BMI increases by unity, the odds of low BMD decreases [OR = 0.9 (0.87–0.94), p < 0.01; for every unit increase in BMI]. AA women had slightly but significantly higher odds of low BMD compared to Whites [OR 1.015 (1.007–1.14), p <0.01 for every unit increase in BMI]. This effect was not observed when Hispanic women were compared to Whites.

There is thus a race-dependent effect of BMI on BMD. With each unit increase in BMI, BMD increases for White women, while a slight but significant decrease in BMD occurs in African American women.

## Background

Osteoporosis is a chronic bone disease characterized by low bone mass and microarchitectural disruption, leading to bone fragility and an increased susceptibility to fractures [1,2]. As a result of increased life expectancy in the population as well as greater public awareness, osteoporosis has evolved from an often overlooked disease entity into a recognized health problem approaching epidemic proportions. In the United States, the disease affects 7.8 million women aged 50 or older accounting for over 1.5 million fractures per year with annual health care expenditures exceeding $13 billion dollars. The National Osteoporosis Foundation reports that close to 22 million women aged 50 or older have low bone mass with projections thru year 2010 and 2020 reaching almost 26 million and over 30 million respectively [3-5]. Third National Health and Nutrition Examination Survey (NHANES III) reported 50%-68% estimated national prevalence of low BMD observed among women aged 50 years or older [6]. The National Osteoporosis Risk Assessment (NORA) study, the largest study of postmenopausal osteoporosis conducted in the United States, with more than 200,000 women aged 50 or older, demonstrated low BMD in almost half of the study population [7]. These numbers, by any standard, portend substantial societal and economic burdens that underscore the importance of disease prevention.

Preventing osteoporosis, a multifactorial disease, resides not only in recognizing its risk factors, but also in identifying potentially modifiable determinants of bone mineral density (BMD), the surrogate measure for osteoporosis [8]. As a result of mounting evidence suggesting beneficial effects on bone, increased body weight has emerged in recent years as a potential modifier of osteoporosis risk [7,9-13]. Likewise, body mass index (BMI), a height-adjusted derivative of body weight, has been accorded the same attention [14-16]. Evidence from the NORA study also demonstrated decreased odds of osteoporosis with increasing BMI and reported BMI as a BMD- protective factor [7]. Among postmenopausal women of Caucasian descent, sufficient evidence has been garnered to suggest that moderate obesity is a protective factor in osteoporosis [7]. In this high risk group, BMI has been shown to positively correlate with BMD [7,17,18]. As BMI increases, BMD increases while the rate of bone loss decreases [19]. The exact physiologic mechanisms that underlie these beneficial effects are unknown but mechanical loading on weight-bearing bones and estrogen synthesis in adipose tissue have been suggested as possible mechanisms [18,20]. Furthermore, studies evaluating the effect of low BMI on BMD have shown unfavourable results. In the *EPIC study*, early postmenopausal women in the lowest tertiles of BMI were shown to have baseline BMD's that were nearly 12 percent lower and experienced over 2-fold increase in bone loss at two years when compared to the highest tertiles of percentage of body fat or BMI [21]. This indicates that a low BMI is an important risk factor for osteoporosis by predisposing to lower peak bone mass and accelerated bone loss. These studies, however, were mainly done on Caucasian women. It is unclear as to what extent; ethnicity plays a role in modifying the effect of BMI on BMD. National and regional surveys in United States demonstrate significant ethnic differences with higher incidence of hip fracture in Caucasian women compared with African American and Mexican American women [22,23]. Similarly, evidence from the NORA study shows increased odds of osteoporosis for Asian and Hispanic women compared to White women as well as decreased odds for African American women. Although prevalence of osteoporosis was higher among Asian and Hispanic women than among Whites, the likelihood of fracture was no different for Hispanics and was in fact lower in Asians [7]. Since, race is an important determinant of body structure and by extension, of body weight and BMI; it seems logical to assume that racial differences in anthropometric constitution may partially explain differences in osteoporosis risk.

To further clarify the beneficial effects of an increasing BMI on BMD, we evaluated the race-dependent effect of BMI on BMD by comparing postmenopausal women from three different racial backgrounds: African American, Hispanic and Caucasian. We hypothesized that, given their inherent advantage of having denser bones compared to Caucasian women, African American or Hispanic women, should have further reduction in osteoporosis risk in the presence of an increasing BMI [24-26].

## Methods

From December 2002 to December 2003, data for this cross-sectional study were obtained by reviewing the clinic records of 3,206 women, aged 50 years or older, screened for osteoporosis at 2 urban centres located in Brooklyn, New York. A standardized data entry sheet was utilized to collect data on patient demographics, and risk factors for osteoporosis. At both sites, bone mineral densities were measured by dual x-ray absorptiometry (DXA) using Hologic^® ^QDR4200. T-scores of non-Caucasian patients were reported by comparing BMD against race-appropriate normative data-bases to adjust for the effect of racial differences. T-scores, as defined by the World Health Organization, is the number of standard deviations above or below the mean BMD value in young adults of the same sex and race [27].

Low BMD was defined as T-score at the lumbar spine <-1. Data was analyzed using SPSS^® ^version 11.5. In comparing two groups for continuous variables, analysis of variance with adjustment for multiple measures employing Benferoni's technique was used with results presented as the mean ± SEM. After adjusting for age, family history of osteoporosis, maternal fracture, smoking, steroid use and sedentary lifestyle, the effect of race on interaction of BMI and BMD was assessed by logistic regression method.

## Results

**Figure 1:**
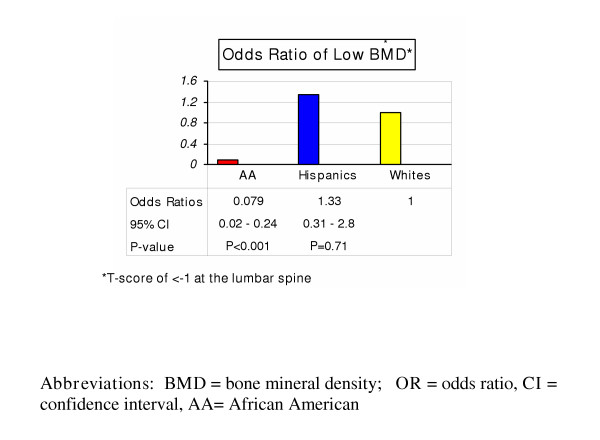
Odds ratio of Low BMD in African American, Hispanic and White Women

**Figure 2:**
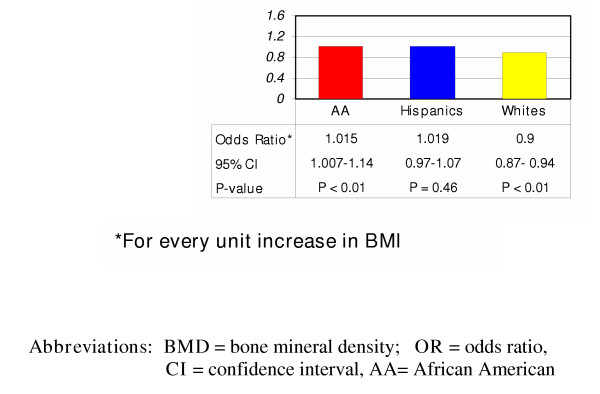
Effect of Race on the Interaction between BMI and BMD

A total of 3,206 women were screened for postmenopausal osteoporosis with their clinic records subsequently reviewed (Table 1). 2,417 (75.4%) were African Americans (AA), 441(13.6%) were Whites, and 348 (10.9%) were Hispanics, reflecting the predominance of AA as the main ethnic group in the community examined. Mean age (± SEM) for the entire cohort was 58.3 ± 0.24. White women were significantly older with a mean age (± SEM) of 60.4 ± 0.62 years while Hispanic women were the youngest at age 56.7 ± 0.67 (p < 0.01). The mean BMI (± SEM) was 30.7 ± 0.87 kg/m^2^; indicating that overall, the group examined was mildly obese. There was no significant difference in BMI among the three groups (p = 0.38). AA women had significantly higher T-scores and BMD (p < 0.01) at both the lumbar spine and hip when compared to the other groups. After adjusting for age, family history of osteoporosis, maternal fracture, smoking, and sedentary lifestyle, AA women had significantly lower odds of having low BMD compared to Whites [Odds ratio (**OR**) = 0.079 (0.03–0.24) (95% CI), p < 0.01]. The odds ratio of low BMD was not significant between White and Hispanic women (Figure 1). On examining the race-dependent effect of an increasing BMI on BMD, for every unit increase in BMI, White women had lower odds of having low BMD [**OR **= 0.9 (0.87–0.94), p < 0.01] while AA women had higher odds of having low BMD [**OR **= 1.015 (1.007–1.14), p < 0.01] when compared to Whites (Figure 2). This effect was not observed when Hispanic women were compared to Whites.

**Table 1:** Demographic characteristics, T-scores, and BMD by race

	**Whites**	**African Americans**	**Hispanics**	**Total**	P*
*N*	441 (13.6%)	2,417 (75.4%)	348 (10.9%)	3,206	
Age (years)	60.4 ± 0.62	58.5 ± 0.28	56.7 ± 0.67	58.6 ± 0.24	<0.01
BMI (kg/m^2^)	27.9 ± 0.30	31.3 ± 1.16	30.2 ± 0.41	30.7 ± 0.87	NS
T-score					
Hip	-1.4 ± 0.06	-0.8 ± 0.04	-0.9 ± 0.06	-0.9 ± 0.03	<0.01
Lumbar spine	-1.3 ± 0.02	-1.4 ± 0.01	-1.5 ± 0.07	-1.4 ± 0.07	NS
BMD (gm/cm^2^)					
Hip	0.777 ± 0.07	0.923 ± 0.01	0.845 ± 0.01	0.899 ± 0.04	<0.01
Lumbar spine	0.905 ± 0.01	0.988 ± 0.01	0.880 ± 0.01	0.965 ± 0.03	<0.01

Using general linear model (GLM), we also assessed BMI, Race and BMI × Race as factors for BMD. Significant main effect was found for BMI (p < 0.01), Race (p < 0.01) and BMI × Race (p < 0.01), indicating that the relationship between BMI and BMD was different between the races.

**Figure 3:**
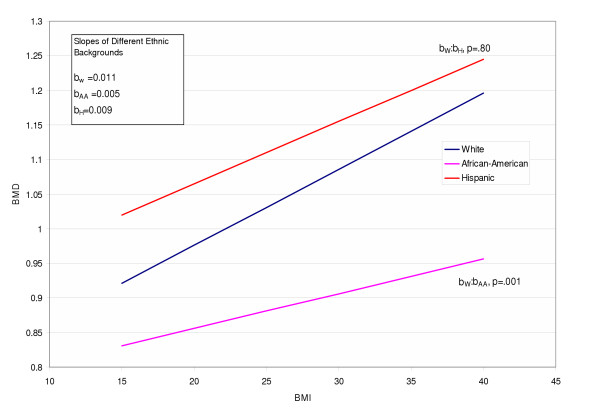
Effect of Race and Obesity on BMD. Bone mineral density (BMD) as it relates to body mass index (BMI). Note the different slopes for each ethnic group. Slopes were significantly different for African American (AA) women and White women p = 0.001. There was no significant difference between Hispanic and White women p = 0.8.

To further examine the linear relationship between BMI and BMD for each race (figure 3), regression parameters were estimated for each race with specific contrasts between each pair. Significant differences were found between Whites and AA women, Hispanic and AA women, but not between Whites and Hispanics (p = 0.8), (Figure 3).

## Discussion

Our study is the first to investigate the effect of race on the interaction of BMI and BMD. The AA women in our study had significantly lower odds of having low BMD, a finding consistent with the results of NORA study [7] and with previous reports that AA women begin menopause with higher BMD and have lower rates of bone loss after menopause [25,26]. On the other hand, Hispanic women were shown to have the same risk of having low BMD as the White women in the study. Although our findings are at variance with the results from NORA study which showed increased odds for osteoporosis for Hispanic women compared with White women, they more closely reflect the observations of similar fracture risk in Hispanic and White women in NORA study [7].

As anticipated, our study demonstrated that in white women, an increasing BMI was associated with slightly lower odds of having a low BMD. This BMD protective effect of increasing BMI and obesity is similar to the findings of other studies including the NORA study [7]. However, when race was introduced as an independent variable to examine its effect on BMD, we were surprised to find that among the AA women, an increase in BMI was associated with a slight albeit significant increase in the odds of having a low BMD. This effect was not observed when Hispanic women were compared to whites. This finding was both unexpected and counterintuitive as an increasing BMI has generally been thought to result in higher BMD. In the NORA study which included more than 18000 minority women, no such correlation between the BMI and BMD was observed in the African American population [7]. However, NORA study also had a predominant Caucasian population (89.7%) and African American women accounted for only 3.9% of study cohort, unlike our study which had predominant African American population.

It is unclear why the protective effect of an increasing BMI is lost in AA women as the present study has not been designed to answer this question. However, the results of our study highlight the need for further studies in this area to confirm our findings and to determine the possible mechanisms that underlie such race-dependent effect of BMI on BMD. Furthermore, previously established risk factors for postmenopausal osteoporosis should be applied with caution to non-white populations as these risk factors were ascertained from studies in which women from minority groups have been underrepresented. As such, studies to establish risk factors for different ethnic groups should be undertaken to attain a more representative estimation of osteoporosis risk that hopefully will lead to an individualized treatment based on ethnic differences.

## Conclusion

Interaction of the BMI and BMD is complex and race is an important modifier of this interaction. Though AA women are less likely to have low BMD than White women, the increase in BMD with BMI seen in Caucasians was not seen in the African Americans. More studies are needed to ascertain why the protective effect of an increasing BMI is lost in AA women and to determine the possible mechanisms that underlie such race-dependent effect of BMI on BMD. Furthermore, previously established risk factors for postmenopausal osteoporosis should be applied to non-white populations with caution as these risk factors were ascertained from studies in which women from minority groups have been underrepresented.

## Abbreviations

**BMI **= body mass index; **BMD **= bone mineral density; **DXA **= dual x-ray absorptiometry, **OR **= odds ratio, **CI **= confidence interval, **AA **= African American

## Competing interests

The author(s) declare that they have no competing interests.

## Authors' contributions

**JPC**, **SA **and **JN **participated in literature search and manuscript preparation, **LJ**, **JS **and **GB **carried out literature search and data collection, **JS, IR, VP, LC, LP, SC, NG, PG **and **RM **participated in the data collection and management. **HG **performed statistical analysis and participated in data presentation, **SIM **conceived of the study, participated in its design, coordination and statistical analysis, helped to draft the manuscript and in final presentation. All authors read and approved the final manuscript.
